# Inhibitory Effects of *Ehretia tinifolia* Extract on the Excessive Oxidative and Inflammatory Responses in Lipopolysaccharide-Stimulated Mouse Kupffer Cells

**DOI:** 10.3390/antiox12101792

**Published:** 2023-09-22

**Authors:** Jae Sung Lim, Sung Ho Lee, Hyosuk Yun, Da Young Lee, Namki Cho, Guijae Yoo, Jeong Uk Choi, Kwang Youl Lee, Tran The Bach, Su-Jin Park, Young-Chang Cho

**Affiliations:** 1 College of Pharmacy and Research Institute of Pharmaceutical Sciences, Chonnam National University, 77 Yongbong-ro, Gwangju 61186, Republic of Korea; dr.jslim7542@gmail.com (J.S.L.); puzim23@gmail.com (S.H.L.); dlekdud0914@naver.com (D.Y.L.); cnamki@jnu.ac.kr (N.C.); cju0667@jnu.ac.kr (J.U.C.); kwanglee@jnu.ac.kr (K.Y.L.); 2 Department of Chemistry, Chonnam National University, Gwangju 61186, Republic of Korea; hslov2aron@hanmail.net; 3 Korea Food Research Institute, 245, Nongsaengmyeong-ro, Iseo-myeon, Wanju-Gun 55365, Republic of Korea; gjyoo@kfri.re.kr; 4 Institute of Ecology and Biological Resources, Vietnam Academy of Science and Technology (VAST), 18 Hoang Quoc Viet, Cau Giay, Ha Noi 122000, Vietnam; tranthebach@yahoo.com; 5 Functional Biomaterial Research Center, Korea Research Institute of Bioscience and Biotechnology, 181 Ipsin-gil, Jeongeup-si 56212, Republic of Korea

**Keywords:** *Ehretia tinifolia*, anti-inflammatory, antioxidant, MAPK, NF-κB, Nrf2

## Abstract

*Ehretia tinifolia* (*E. tinifolia*) L., an evergreen tree with substantial biological activity, including antioxidant and anti-inflammatory effects, has been used in many herbal and traditional medicines. To elucidate its antioxidant and anti-inflammatory activity and the underlying mechanisms, we applied a methanol extract of *E. tinifolia* (ETME) to lipopolysaccharide (LPS)-stimulated mouse immortalized Kupffer cells. ETME suppressed the LPS-induced increase in nitric oxide, a mediator for oxidative stress and inflammation, and restored LPS-mediated depletion of total glutathione level by stabilizing antioxidative nuclear factor erythroid 2-related factor 2 (Nrf2) and the subsequent increase in heme oxygenase-1 levels. Furthermore, ETME inhibited the LPS-induced production of pro-inflammatory cytokines, including tumor necrosis factor-α, interleukin (IL)-1β, and IL-6. The inhibitory effects of ETME on pro-inflammatory responses were regulated by ETME-mediated dephosphorylation of mitogen-activated protein kinases (MAPKs: p38, p44/p42, and stress-associated protein kinase/c-Jun N-terminal kinase) and inhibition of nuclear localization of nuclear factor kappa B (NF-κB). These results suggest that ETME is a possible candidate for protecting Kupffer cells from LPS-mediated oxidative stress and excessive inflammatory responses by activating antioxidant Nrf2/HO-1 and inhibiting pro-inflammatory NF-κB and MAPKs, respectively.

## 1. Introduction

Inflammation is a physiological response by the immune system to harmful stimuli, playing an important role in the body’s defense mechanisms. However, uncontrolled inflammation can contribute to the development of various diseases including autoimmune diseases, metabolic syndrome, neurodegenerative diseases, cancers, and cardiovascular diseases [1]. Although non-steroidal anti-inflammatory drugs (NSAIDs) are mostly used for the treatment of patients suffering from pain and inflammatory disorders, NSAIDs have severe adverse effects including the gastrointestinal toxicities, cardiovascular risks, renal injuries, hypertension, and hepatotoxicity [2]. Therefore, there is a need to discover better anti-inflammatory drugs.

Natural products have massive structural and chemical diversity that cannot be matched by any synthetic libraries of small molecules. They continue to encourage novel discoveries in the fields of chemistry, biology, and medicine. Moreover, natural products are evolutionarily optimized as drug-like molecules, making them the best sources of drugs and drug leads [3]. Given their extensive history of use, natural products have shown promise in the treatment of inflammatory disorders and even certain types of cancers [4,5,6]. The extracts of whole plants [7], fruit [8], leaves [9], and plant-based formulations [10] have considerable antioxidant and anti-inflammatory effects [11,12]. Harnessing the therapeutic properties of natural products could lead to the development of safer and more effective anti-inflammatory therapies.

*Ehretia tinifolia* (*E. tinifolia*) L. is a plant species in the family Boraginaceae. It is also known as a ‘pinguica’ and a tree with small, round, fragrant, and sweet yellow fruit widely used as food in Mexico and the United States [13,14]. It has been used as a traditional medicine for the treatment of urinary track disorder by reducing uric acid. Also, the bark of this plant has been used for wound healing, and the flowers and leaves have been used to treat bloody vomiting [15,16,17,18]. Recently, it was reported that the fruit of *E. tinifolia* has antioxidant effects [19], which are associated with the phenol content of its polar organic extracts [20]. Recent examination of the chemical composition and biological activity of the fruit revealed rosmarinic acid as one of the main active components [21]. This hydroxylated compound occurs in many herbal plants and has been extensively studied for its antioxidant and anti-inflammatory activities [22,23]. However, other parts such as leaf, flower, and branch, except for the fruit of *E. tinifolia,* have not been studied yet.

To elucidate the antioxidant and anti-inflammatory effects of *E. tinifolia*, we tested the effects of a methanol extract of the leaf, flower, and branch of *E. tinifolia* (ETME) on lipopolysaccharide (LPS)-stimulated immortalized mouse Kupffer cells (ImKCs). Kupffer cells, macrophages in the liver derived from monocytes in the bloodstream, play an important role in clearing foreign substances, including immune complexes, bacterial components, and endotoxins, from the portal circulation [24]. They are activated by numerous molecules, including bacterial endotoxins such as LPS. When activated, they secrete various pro-inflammatory cytokines, including tumor necrosis factor (TNF)-α and several types of interleukins (ILs), which act as inflammatory cytokines, eliminating aberrant toxic or foreign substances and initiating healing [25].

Our findings reveal that ETME exerts anti-inflammatory effects by inhibiting the mitogen-activated protein kinase (MAPK) and nuclear factor kappa-light-chain-enhancer of activated B cells (NF-κB) signaling pathways and antioxidant activity by inducing the nuclear factor erythroid 2-related factor 2 (Nrf2)/heme oxygenase 1 (HO-1) signaling pathway. This fruit has the potential to treat liver diseases such as liver fibrosis.

## 2. Materials and Methods

### 2.1. Methanol Extraction of E. tinifolia

*E. tinifolia* plant material (whole leaves, flowers, and branches of the plant) was collected in the Da Chais community, Lac Duong district, Lam Dong province, Vietnam, by Dr. Tran The Bach (Institute of Ecology and Biological Resources, Hanoi, Vietnam), who also verified the identification. Voucher specimens (KRIB41338 and VK4876) were deposited in the herbarium of the Korea Research Institute of Bioscience and Biotechnology (Daejeon, Republic of Korea). A total of 50 g of the plant material was dried in the shade, powdered, added to 1 L of high-performance liquid chromatography (HPLC) grade methanol, and extracted via 30 cycles of ultrasonication (40 kHz, 1500 W, 15 min per cycle, with a 120 min rest between cycles) at room temperature, using an ultrasonic extractor (SDN-900H; SD-ULTRASONIC Co., Ltd., Seoul, Republic of Korea). This mixture was then filtered and dried at 40 °C under reduced pressure to obtain ETME.

### 2.2. Ultra-HPLC-Quadrupole Time-of-Flight Mass Spectrometry (UPLC-Q-TOF-MS) Analysis

The dried sample extracts (1 mg/mL) were dissolved in aqueous methanol for UPLC-Q-TOF-MS analysis using an LTQ IT mass spectrometer (Thermo Fisher Scientific, Waltham, MA, USA) equipped with an electrospray interface, RS column compartment, and RS pump (Dionex Corporation, Sunnyvale, CA, USA). Chromatographic separation was performed on a Thermo Fisher Scientific Syncronis C18 HPLC column (100 × 2.1 mm internal diameter; 1.7 μm particle size) with an injection volume of 2 μL. The mobile phase comprised water (solvent A) with 0.1% formic acid (*v*/*v*) and acetonitrile (solvent B) with 0.1% formic acid (*v*/*v*) at a flow rate of 0.4 mL/min; the column temperature was 35 °C. The solvent gradient was 5% B for 1 min, increased to 100% B for 20 min, maintained for 2.5 min, decreased to 5% B for 1 min, and maintained at 10% B for the final 3 min. The total run time was 25 min. The mass spectra and photodiode array range in the negative mode were tuned for *m*/*z* 50–1200 and 200–600 nm, respectively. The collision voltage was 4 V, and the source voltage was ±1 kV.

### 2.3. Radical Scavenging (DPPH) Assay

High-concentration stocks of ETME (100 mg/mL), quercetin (100 mg/mL), and rosmarinic acid (40 mg/mL) were prepared in DMSO solution, and then ETME and rosmarinic acid were prepared into each treatment concentration [ETME: 10, 20, 40, 80, and 160 μg/mL, rosmarinic acid: 5 μg/mL (13.88 μM), 10 μg/mL (27.75 μM), and 20 μg/mL (55.51 μM)) by half serial dilution using 100% methanol. And the quercetin was diluted in 100% methanol [10 μg/mL (33.09 μM) and 30 μg/mL (99.26 μM)]. Then, 100 µL of each diluent was transferred into 96-well plates. Afterwards, 100 µL of 0.2 mM DPPH solution was added and incubated for 30 min in a 37 °C incubator. After incubation, absorbance was measured at 517 nm using a Synergy H1 Hybrid Microplate Reader. The DPPH inhibition was expressed as % using the OD value of the experimental group and the value of 100% methanol solution (as a negative control). Quercetin (Sigma-Aldrich, St. Louis, MO, USA) was used as a positive control. The calculation formula of DPPH inhibition is as follows:

DPPH inhibition (%) = (OD_517nm Sample_ − OD_517 Methanol_)/OD_517 Methanol_ × 100

### 2.4. Cell Culture

LPS-stimulated ImKCs (#SCC119, Sigma-Aldrich, St. Louis, MO, USA) were cultured in Dulbecco’s modified Eagle’s medium (DMEM; #LM0001-05; WELGENE Inc., Gyeongsan, Republic of Korea) supplemented with 10% fetal bovine serum (16000044; Gibco, Waltham, MA, USA), 100 IU/mL penicillin, and 100 µg/mL streptomycin (#30-002-cl; Corning Inc., Corning, NY, USA) at 37 °C in a CO_2_ incubator. Dried extract of ETME was dissolved in 100% DMSO (stock concentration of ETME: 160 mg/mL). When cells were treated with ETME, it was diluted in the cell culture medium and the concentration of DMSO was equally controlled at 0.1%, a non-toxic concentration in cells, in all experimental groups.

### 2.5. Cell Viability Assay

The ImKCs were seeded into 96-well plates (4 × 10^4^/well). The cells were cultured with various concentrations (10, 20, 40, 80, or 160 µg/mL) of ETME for 24 h. Cell viability was measured using an EZ-Cytox cell viability assay kit (#EZ-1000; DoGenBio, Seoul, Republic of Korea). Briefly, the cells were incubated with the EZ-Cytox solution (containing a water-soluble tetrazolium salt) for 2 h at 37 °C. Cell viability was determined by quantifying the metabolic conversion of the tetrazolium salt to formazan dye. The absorbance of the supernatant was measured at 450 nm using a microplate reader (Synergy HTX; BioTek Instruments, Inc., Winooski, VT, USA). Cell viability was calculated using the following equation:

Cell viability (%) = (OD_420 Experimental group_ − OD_420 Background control_)/(OD_420 Untreated group_ − OD_420 Background control_) × 100

where OD_420 Background control_ represented the optical density of EZ-Cytox solution with the medium without a cell.

### 2.6. Lactate Dehydrogenase (LDH) Release Assay

Release assays for lactate dehydrogenase (LDH, an indicator of cell damage) were performed as previously described [6]. Cytotoxicity was determined using an LDH assay kit (#DG-LDH500; DoGenBio) according to the manufacturer’s protocol. The cells were seeded in triplicate into 12-well plates (reaching 60% confluence on day 0), then pretreated with ETME (10, 20, 40, 80, or 160 μg/mL) for 2 days. Thereafter, the cells were treated with LPS (1 µg/mL) for 24 h in a CO_2_ incubator. The culture supernatants were collected, and 10 μL of the supernatant was transferred into 96-well plates, followed by incubation with 100 μL of LDH solution at room temperature in the dark for 30 min. Optical density (OD) was measured at 450 nm using a Synergy HTX microplate reader (BioTek Instruments). Cytotoxicity was calculated as previously described [6] using the following equation:

Cytotoxicity (%) = [(OD_450 Experimental control_ − OD_450 Background control_) − (OD_450 LDH_min control_ − OD_450 Background control_)]/[(OD_450 LDH_max control_ − OD_450 Background control_) − (OD_450 LDH_min control_ − OD_450 Background control_)] × 100

where OD_450 Background control_ represented the optical density of LDH in the complete medium, OD_450 LHD_max control_ represented the maximum amount of LDH released by lysis from the cells, and OD_450 LDH_min control_ represented the minimum amount of LDH released by cells that had died naturally. For the “Volume” control, we added lysis solution to the complete medium.

### 2.7. Glutathione (GSH) Assay

A GSH assay kit (#703002; Cayman Chemical Company, Ann Arbor, MI, USA) was used to determine the amount of GSH in the cells, according to the manufacturer’s guidelines. The ImKCs were seeded into 6-well plates (1.0 × 10^6^ cells/well). Cells were pretreated with various concentrations of ETME (20, 40, 80, or 160 µg/mL) for 2 h, then stimulated with LPS (1 µg/mL) for 10 min. Thereafter, the cells were washed with cold phosphate-buffered saline (PBS) and collected by centrifugation. The cell pellet was sonicated in 50 mM cold MES buffer (from the GSH assay kit) and centrifuged at 10,000× *g* for 15 min at 4 °C. Next, the supernatant was collected in a new tube, and 1 M 2-vinylpyridine (#13229-2; Sigma-Aldrich) was added at a volume one-tenth that of the supernatant. The samples and standards were transferred into 96-well plates, and the assay cocktail mixture (from the GSH assay kit) was added to each sample at four times the volume of the sample. The plate was then incubated on an orbital shaker for 25 min in the dark, and absorbance was measured using a Synergy HTX microplate reader (BioTek Instruments) at 410 nm. We calculated GSH as follows. A linear regression equation (Y = βX + α; Y: absorbance, β: slope, X: sample’s concentration, α: intercept) with the concentration of the standard reagent (glutathione) in each experimental analysis as the *X*-axis and the absorbance (or optical density, OD) value as the *Y*-axis was calculated, and linear regression (r), slope, and Y-intercept were obtained. The obtained OD value in each experiment was substituted into a linear regression equation to calculate each experimental value. And its average value was used as the titer of the experimental group. When using a diluted sample, the sample’s dilution factor was reflected in the calculation. Moreover, if the linear regression (r) was 0.990 or more, the linear regression equation was valid.

### 2.8. Measurement of Nitric Oxide (NO) Production

The ImKCs were seeded into 12-well plates (4.0 × 10^5^ cells). The cells were pretreated with various concentrations of ETME (10, 20, 40, 80, or 160 μg/mL) for 2 h, then stimulated with LPS (1 µg/mL) for 24 h. Thereafter, the cell supernatants (100 µL) were transferred into new 96-well plates, and 100 µL Griess reagent (1% sulfanilamide, 0.1% N-1-naphthylethylenediamine dihydrochloride, and 2.5% phosphoric acid) was added. Absorbance at 540 nm was measured using a Synergy HTX microplate reader (BioTek Instruments). We calculated NO as follows. A linear regression equation (Y = βX + α; Y: absorbance, β: slope, X: sample’s concentration, α: intercept) with the concentration of the standard reagent (nitrite) in each experimental analysis as the *X*-axis and the absorbance (or optical density, OD) value as the *Y*-axis was calculated, and linear regression (r), slope, and Y-intercept were obtained. The obtained OD value in each experiment was substituted into a linear regression equation to calculate each experimental value. And its average value was used as the titer of the experimental group. When using a diluted sample, the sample’s dilution factor was reflected in the calculation. Moreover, if the linear regression (r) was 0.990 or more, the linear regression equation was valid.

### 2.9. Western Blotting

The cells were pretreated with various concentrations of ETME (20, 40, 80, or 160 µg/mL) for 2 h, then stimulated with LPS (1 µg/mL) for 15 min or 24 h. The cells were lysed with rapid immunoprecipitation assay (RIPA) buffer (#RC2002-050-00; Biosesang, Seongnam, Republic of Korea) containing a protease inhibitor cocktail and phosphatase inhibitor cocktails II and III (#P8340, #P5726, and #P0044, respectively; Sigma-Aldrich). The whole-cell lysate was denatured in 5× sodium dodecyl sulfate (SDS) sample buffer at 95 °C for 10 min, separated by SDS-polyacrylamide gel electrophoresis, then transferred onto nitrocellulose membranes. To block nonspecific binding, the membranes were incubated in 5% nonfat dry milk (#SKI500, LPS Solution, Daejeon, Republic of Korea) in Tris-buffered saline and Tween-20 (25 mM Tris-HCl pH 8.0, 125 mM NaCl, and 0.1% Tween-20) for 1 h at room temperature. The membranes were incubated with anti-inducible nitric oxide synthase (anti-iNOS; #610332; BD Biosciences, San Diego, CA, USA), anti-β-actin, anti-p38, anti-p44/42 (#sc-47778, #sc-7972, and #sc-514302, respectively; Santa Cruz Biotechnology Inc., Dallas, TX, USA), anti-stress-associated protein kinase/c-Jun N-terminal kinase (anti-SAPK/JNK), anti-phospho-SAPK/JNK, anti-phospho-p38, and anti-phospho-p44/42 (#9252, #9251, #9211, and #9101, respectively; Cell Signaling Technology, Danvers, MA, USA) antibodies at 4 °C overnight, then incubated with horseradish peroxidase-conjugated (HRP) secondary antibodies (Cell Signaling Technology) for 1 h at room temperature. Western blotting substrate for enhanced chemiluminescence (#BWP0200; Biomax, Seoul, Republic of Korea) was used to detect HRP-conjugated secondary antibodies. Protein expression was analyzed using a ChemiDoc imaging system (Amersham Imager 680; GE Healthcare, Chicago, IL, USA) and quantified using ImageJ (National Institutes of Health, Bethesda, MD, USA). The quantification graph of protein expression was basically calculated based on the obtained values from each protein band. First, the protein bands were quantified with the specific length and height of each protein band under the same conditions in quantitative software. Then, the value of target proteins was normalized by the value of internal control (β-actin, GAPDH, and Lamin B1). Finally, the normalized values were graphed and expressed as protein expression. In the case of the phospho-form, proteins were normalized by the values of total protein based on the values of β-actin, and the normalized values were graphed.

### 2.10. Enzyme-Linked Immunosorbent Assay (ELISA)

The ImKCs were seeded into 12-well plates (4.0 × 10^5^ cells/well). The cells were pretreated with various concentrations of ETME (20, 40, 80, or 160 µg/mL) for 2 h, then stimulated with LPS (1 µg/mL) for 24 h. The expression of the indicated cytokines in cell supernatants was measured using an ELISA kit. Purified anti-IL-6 (#554400; BD Pharmingen, San Diego, CA, USA), anti-IL-1β, and anti-TNF-α (#14-7012-85 and #14-7423-85, respectively; Thermo Fisher Scientific Inc., Waltham, MA, USA) antibodies were coated onto 96-well plates and incubated overnight at 4 °C. The plates were washed three times with 0.05% Tween-20 in PBS, then incubated with 1% bovine serum albumin (BSA) in PBS for 1 h at room temperature. The supernatants and standard solutions were incubated for 2 h at room temperature and washed three times. Thereafter, the plate was incubated with detection antibodies for 1 h at room temperature and washed three times, then incubated with streptavidin-conjugated alkaline phosphatase (AKP; #554065; BD Pharmingen) solution for 30 min at room temperature, and washed five times. Finally, the plate was incubated in the dark with a substrate buffer (pH 9.8), comprising 10% diethanolamine, 0.1% MgCl_2_·6H_2_O, 0.2% NaN_3_ (#3032-4400, #5503-44, and #7530-4105, respectively; Daejung Chemicals & Metals Co., Siheung, Republic of Korea), and 4-nitrophenyl phosphate (#N2765; Sigma-Aldrich). Subsequently, 1 N NaOH was added to stop the reaction. Absorbance at 450 nm was measured using a Synergy HTX microplate reader (BioTek Instruments). We calculated IL-6, IL-1β, and TNF-α as follows. A linear regression equation (Y = βX + α; Y: absorbance, β: slope, X: sample’s concentration, α: intercept) with the concentration of the standard reagents (IL-6, IL-1β, and TNF-α) in each experimental analysis as the *X*-axis and the absorbance (or optical density, OD) value as the *Y*-axis was calculated, and linear regression (r), slope, and Y-intercept were obtained. The obtained OD value in each experiment was substituted into a linear regression equation to calculate each experimental value. And its average value was used as the titer of the experimental group. When using a diluted sample, the sample’s dilution factor was reflected in the calculation. Moreover, if the linear regression (r) was 0.990 or more, the linear regression equation was valid.

### 2.11. Fractionation of Nuclear and Cytoplasm Proteins

Nuclear and cytoplasmic fractionation was performed according to the manufacturer’s protocol using the NE-PER Nuclear and Cytoplasmic Extraction Reagent Kit (#78833; Thermo Fisher Scientific Inc.). Briefly, ImKCs were seeded into 6-well plates (2.0 × 10^6^ cells/well), pretreated with 160 μg/mL of ETME for 2 h, then stimulated with LPS (1 µg/mL) for 10 min. The cells were washed twice with ice-cold PBS and lysed with 200 μL cytoplasmic lysis buffer (from the fractionation kit) on ice for 10 min. The lysates were centrifuged at maximum speed for 10 min at 4 °C, and the supernatants were collected to obtain the cytoplasmic fraction. Next, the pellet was resuspended in 100 μL nuclear extraction buffer (from the fractionation kit) on ice for 40 min and vortexed every 10 min. After centrifugation for 10 min at 4 °C, the supernatant was collected to obtain the nuclear fraction. Western blotting was performed using anti-GAPDH (a cytoplasmic marker), anti-NF-κB p65 (#sc-365062 and #sc-8008, respectively; Santa Cruz Biotechnology Inc.), anti-Lamin B1 (a nuclear marker), and anti-inhibitor of κB (IκB) (#9242 and #9242, respectively; Cell Signaling Technology) antibodies.

### 2.12. Immunofluorescence Staining

The ImKCs were seeded into 24-well plates (2.0 × 10^5^ cells/well) with coverslips. The cells were pretreated with 160 μg/mL ETME for 2 h, then stimulated with LPS (1 µg/mL) for 10 min. The cells were fixed with a 4% paraformaldehyde solution (#PC2031-050-00; Biosesang) and permeabilized with 0.1% Triton X-100 (#T8787; Sigma-Aldrich) for 10 min at room temperature. Next, the cells were blocked with PBS containing 1% BSA at room temperature for 1 h, then incubated with an anti-NF-κB antibody at 4 °C overnight. Thereafter, the cells were incubated with secondary antibodies in the dark for 1 h. Finally, the cells were fixed onto glass slides using a mounting solution (#S36936; Thermo Fisher Scientific Inc.), and fluorescent images were captured using a confocal microscope (Nikon AX R; Nikon Instruments, Tokyo, Japan).

### 2.13. Statistical Analysis

The data are presented as the mean ± standard error of the mean (SEM) and were analyzed using Prism v8.0 (GraphPad Inc., San Diego, CA, USA). The results were analyzed using the nonparametric Mann–Whitney U test, with *p* < 0.05 considered statistically significant. All experiments were performed in triplicates.

## 3. Results

### 3.1. Rosmarinic Acid Is a Major Consituent of ETME

**Figure 1:**
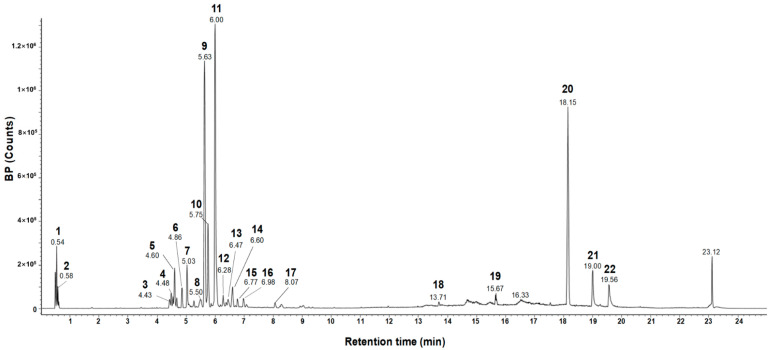
UPLC-Q-TOF-MS spectrometry analysis of ETME.

Previous studies revealed that rosmarinic acid is the major component of *E. tinifolia* through a phytochemical assay-guided fractionation [21] and it has antioxidant and anti-inflammatory properties [22,23]. Before investigating the effects of ETME on oxidative stress and inflammation, an evaluation of the major components in ETME that exhibit antioxidant and anti-inflammatory effects was performed. The UPLC-Q-TOF-MS and the total ion chromatogram (TIC) of the MeOH extract are shown in Figure 1. The analysis revealed the presence of 22 phytochemicals belonging to various subclasses such as oligosaccharides, flavonoids, phenolic acids, and lignans, as detailed in Table 1. One of the most abundant compounds in ETME observed by the mass analysis was the predominant precursor ion at *m*/*z* 359, which can be attributed to rosmarinic acid as confirmed by a standard product retention time (retention time of 11.30 min; Supplementary Figure S1). Compound methyl rosmarinate was identified at *m*/*z* 374, as previously reported in *E. tinifolia* ethyl acetate portion [26], and was known for its antioxidant and antifungal activities [22,27]. For the identification of the remaining compounds, the *m*/*z* values of the molecular ion [M-H]^−^ and [M + HCOO]^−^ were compared with the Waters Unifi Software Traditional Medicine Library (Waters Corporation, Milford, MA, USA) and corroborated from the previously reported literature [28,29,30,31,32,33,34,35,36,37,38,39,40,41,42]. Among these compounds, schisantherin A, kaempferol-3,7-diglucoside, procyanidin A2, and tocopherol exert anti-inflammatory and antioxidant activity and promote reproductive functions [43,44,45,46], which explain the potent antioxidant and anti-inflammatory activity observed in *E. tinifolia*. Through UPLC-Q-TOF-MS spectral analysis, it is evident that rosmarinic acid comprises a larger proportion relative to other components. Therefore, it can be inferred that rosmarinic acid predominantly exerts its influence on antioxidant and anti-inflammatory effects. Herein, an additional experiment was performed to assess ETME’s antioxidant properties by measuring rosmarinic acid-mediated antioxidant activity. Rosmarinic acid showed a dose-dependent increase in DPPH inhibition and showed a high degree of inhibition at 20 μg/mL similar to those of quercetin 10 μg/mL, a well-known antioxidant molecule, indicating that ETME might exhibit antioxidant properties (Supplementary Figure S2). These data prompted us to study the antioxidant and anti-inflammatory effects and underlying regulatory mechanisms of action of ETME in ImKCs.

### 3.2. ETME Exerted Antioxidant Properties under Non-Cytotoxic Concentrations

**Figure 2:**
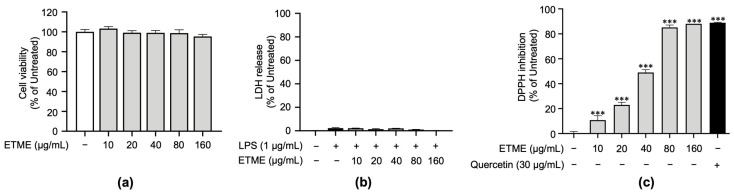
Effects of ETME on cell viability of ImKCs and free radical scavenging activity. (**a**) ImKCs were treated with various concentrations of ETME for 24 h. Cell viability was then measured using the EZ-Cytox reagent and compared with that of the untreated group. (**b**) The ImKCs were treated with LPS (1 μg/mL) in the presence of ETME (10, 20, 40, 80, or 160 μg/mL) for 24 h. The supernatants were collected and analyzed using the lactate dehydrogenase (LDH) assay kit. Cells treated with lysis buffer were used as a positive control (100% LDH release). (**c**) The antioxidant effect of ETME was analyzed by DPPH assay. Quercetin was used as a positive control. The data are the mean ± standard error of the mean (SEM) of three independent experiments. Differences between groups were analyzed using the Mann–Whitney U test. ETME: *E. tinifolia* methanol extract; DPPH: 2,2-diphenyl-1-picryhydrazyl. *** *p* < 0.001 vs. untreated group.

To verify non-cytotoxic concentrations of ETME on ImKCs’ cell viability, a cell viability assay based on tetrazolium conversion and an LDH assay were carried out in the absence or presence of LPS. Both assays revealed that ETME showed no significant cytotoxicity in ImKCs at various concentrations of up to 160 μg/mL (Figure 2a,b). Therefore, subsequent experiments were conducted at ETME concentrations of up to 160 μg/mL. Next, to examine whether ETME has antioxidant properties, DPPH assay was conducted with ETME and quercetin. ETME exhibited a dose-dependent increase in DPPH inhibition and showed a similar effect at 160 μg/mL with that of quercetin, indicating that ETME has antioxidant properties (Figure 2c).

### 3.3. ETME Rescued GSH Levels and Inhibited NO Production

**Figure 3:**
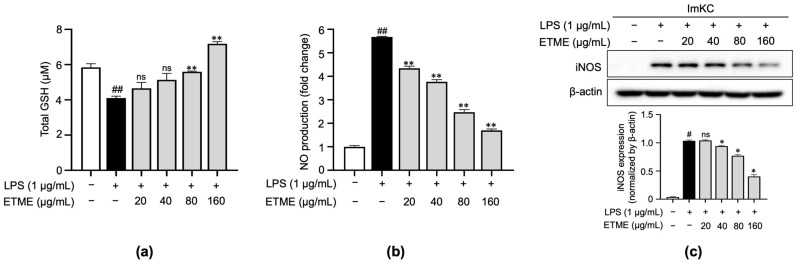
Effects of ETME on total glutathione (GSH), nitric oxide (NO) production, and inducible nitric oxide synthase (iNOS) expression in ImKCs. The ImKCs were treated with LPS (1 μg/mL) at the indicated concentration of ETME (20, 40, 80, or 160 μg/mL) for 24 h. (**a**) ImKCs were pretreated with ETME (20, 40, 80, or 160 µg/mL) for 2 h, then stimulated with LPS (1 μg/mL) for 10 min. Total GSH was measured using a GSH assay kit. (**b**) NO production in the culture supernatant was measured using a Griess assay. NO secretion was calculated using a standard curve of nitrite standard-solution concentrations. (**c**) Inducible nitric oxide synthase (iNOS) expression was detected by Western blotting. β-actin was used as a loading control. Protein expression was normalized to that of β-actin. The data are the mean ± SEM of three independent experiments. Differences between groups were analyzed using the Mann–Whitney U test. # *p* < 0.05, ## *p* < 0.01 vs. LPS-untreated group; * *p* < 0.05, ** *p* < 0.01 vs. LPS-treated group; ns: not significant.

Based on the radical scavenging effect of ETME, the antioxidant property of ETME was evaluated by measuring total reduced GSH levels in LPS-treated ImKCs. As shown in Figure 3a, the LPS-mediated increase in oxidative stress was detected by the decrease in total reduced GSH levels in LPS-treated ImKCs. The LPS-mediated decrease in total reduced GSH levels was recovered by ETME treatment (Figure 3a), indicating that ETME has antioxidant properties in ImKCs. The production of NO, a well-known mediator for oxidative stress and inflammation [47], was significantly induced in LPS-treated ImKCs. ETME suppressed LPS-induced NO production in a dose-dependent manner (Figure 3b). The LPS-stimulated ImKCs express pro-inflammatory enzymes, such as iNOS, which play an important role in NO production [48]. Consistent with NO production, LPS-induced iNOS expression was significantly suppressed by ETME in ImKCs (Figure 3c), implying that ETME alleviates NO production in LPS-stimulated ImKCs through the transcriptional inhibition of iNOS.

### 3.4. ETME Inhibited Pro-Inflammatory Cytokine Production

**Figure 4:**
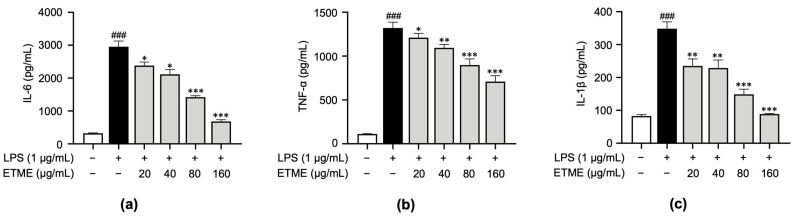
Inhibitory effects of ETME on pro-inflammatory cytokine production. ImKCs were treated with LPS in the presence of ETME (0, 20, 40, 80, or 160 μg/mL). (**a**–**c**) After stimulation for 24 h, the culture supernatants were collected and analyzed for interleukin (IL)-6, tumor necrosis factor (TNF)-α, and IL-1β production via an enzyme-linked immunosorbent assay (ELISA). The data presented are the mean ± SEM of three independent experiments. Differences between groups were analyzed using the Mann–Whitney U test. ### *p* < 0.001 vs. LPS-untreated group; * *p* < 0.05, ** *p* < 0.01, *** *p* < 0.001 vs. LPS-treated group.

To investigate whether ETME inhibits the secretion of pro-inflammatory cytokines, including TNF-α, IL-1β, and IL-6, in ImKCs, supernatants were collected following the treatment of ETME in the presence of LPS and subjected to an ELISA. While the secretion of pro-inflammatory cytokines was markedly induced in LPS-stimulated ImKCs, the ETME treatment inhibited secretory levels of those cytokines in a dose-dependent manner (Figure 4), revealing the anti-inflammatory effects of ETME in LPS-stimulated ImKCs.

### 3.5. ETME Exerted Anti-Inflammatory Activity by Activating HO-1

**Figure 5:**
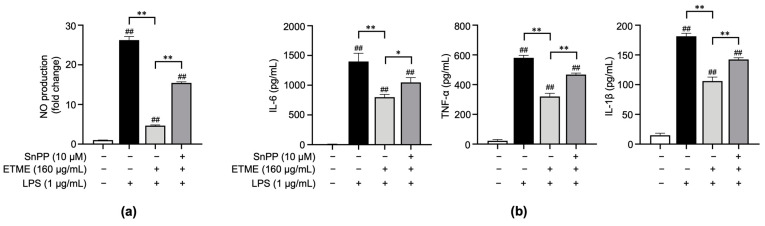
The protective action of ETME on NO production and pro-inflammatory cytokine production is heme oxygenase-1 (HO-1)-dependent. ImKCs were treated with the indicated combinations of LPS (1 μg/mL), ETME (160 μg/mL), and Sn protoporphyrin (SnPP, 10 μM) for 24 h. (**a**) NO production in the culture supernatant was measured using a Griess assay. NO secretion was calculated using a standard curve of nitrite standard-solution concentration. (**b**) The culture supernatants were collected and analyzed for IL-6, TNF-α, and IL-1β production via an ELISA. The data presented are the mean ± SEM of three independent experiments. Differences between groups were analyzed using the Mann–Whitney U test. ## *p* < 0.01 vs. LPS-untreated group; * *p* < 0.05, ** *p* < 0.01 between paired groups.

The results shown in Figure 2, Figure 3 and Figure 4 indicate that ETME alleviates LPS-induced oxidative stress and pro-inflammatory responses. To clarify whether ETME regulates oxidative stress through the activation of antioxidant signaling molecules, Sn protoporphyrin (SnPP), a well-known HO-1 inhibitor [49], was co-treated with ETME in LPS-stimulated ImKCs. The ETME-mediated inhibition of NO production was significantly attenuated by SnPP treatment (Figure 5a). These results revealed that the ETME-mediated inhibition of NO is regulated by the ETME-induced increase in antioxidant expression. To further investigate whether the increase in antioxidant HO-1 expression is involved in the anti-inflammatory effect of ETME, the secretory levels of pro-inflammatory cytokines were measured in the same supernatants. SnPP treatment partially abolished the inhibitory effects of ETME on pro-inflammatory cytokine production (Figure 5b), suggesting that the ETME-mediated increase in HO-1 expression leads to its antioxidant and anti-inflammatory effects in LPS-stimulated ImKCs.

### 3.6. ETME Increased HO-1 and Nrf2 Expression Thereby Promoting Its Antioxidant Activity

**Figure 6:**
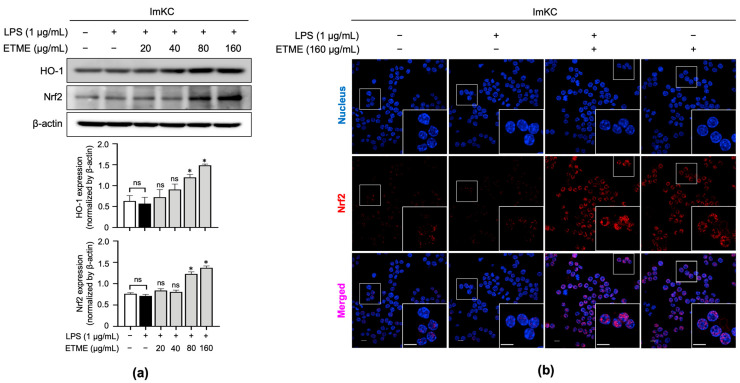
Effects of ETME on nuclear factor erythroid 2-related factor 2 (Nrf2) nuclear localization and HO-1 expression. (**a**) ImKCs were treated with LPS (1 μg/mL) in the presence of ETME (0, 24, 40, 80, or 160 μg/mL) for 24 h, after which total protein was extracted. Western blotting was used to detect HO-1 and Nrf2 expression, with β-actin as a loading control. Protein expression was normalized to that of each loading control. The data presented are the mean ± SEM of three independent experiments. (**b**) The nuclear translocation of Nrf2 (red) in cells was analyzed by confocal microscopy. Nuclei were stained with 4′,6-diamidino-2-phenylindole (blue). Scale bar, 10 μm. Differences between groups were analyzed using the Mann–Whitney U test. * *p* < 0.05 vs. LPS-treated group; ns: not significant.

Nrf2 is a transcription factor responsible for regulating the expression of genes, such as HO-1, involved in cellular redox balance and protective antioxidant systems [50]. Nrf2 pathway activation is thus a possible explanation for HO-1 induction. As shown in Figure 6a, ETME significantly induced expression levels of Nrf2 at high concentrations (80 and 160 μg/mL) compared to LPS-stimulated ImKCs. Further investigation was conducted to evaluate Nrf2 accumulation in the nucleus. Immunofluorescence data showed that ETME increased nuclear Nrf2 expression (Figure 6b). These results indicate that ETME exhibits antioxidant activity via the nuclear accumulation of Nrf2 protein and subsequent increase in HO-1 expression.

### 3.7. ETME Inhibited NF-κB/p65 Nuclear Translocation

**Figure 7:**
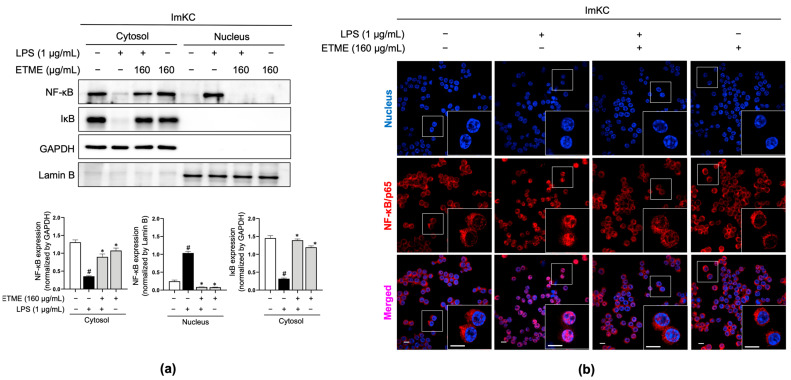
Inhibitory effects of ETME on nuclear factor kappa-light-chain-enhancer of activated B cells (NF-κB)/p65 nuclear translocation. ImKCs were pretreated with ETME (160 μg/mL) for 2 h, then stimulated with LPS (1 μg/mL) for 15 min. (**a**) NF-κB/p65 and inhibitor of κB (IκB) expression in cytosolic and nuclear extracted-protein samples was detected by Western blotting. Glyceraldehyde 3-phosphate dehydrogenase (GAPDH) was used as a cytosolic loading control and Lamin B as a nuclear loading control. Protein expression was normalized to that of each loading control. The data presented are the mean ± SEM of three independent experiments. Differences between groups were analyzed using the Mann–Whitney U test. # *p* < 0.05 vs. LPS-untreated group; * *p* < 0.05 vs. LPS-treated group. (**b**) The nuclear translocation of NF-κB/p65 (red) in cells was analyzed by confocal microscopy. Nuclei were stained with 4′,6-diamidino-2-phenylindole (blue). Scale bar, 10 μm.

The NF-κB signaling pathway, a major regulator of inflammatory responses in macrophages upon LPS stimulation [51,52], is activated primarily by the nuclear localization of NF-κB/p65 after IκB degradation [52]. Total proteins were fractionated into cytosolic and nucleus fractions and subjected to immunoblotting analysis to detect IκB and NF-κB/p65. In the cytosolic fraction, LPS stimulation induced IκB degradation, whereas ETME reversed the LPS-induced IκB degradation (Figure 7a). Considering NF-κB/p65 levels in both fractions, ETME inhibited the LPS-induced translocation of NF-κB/p65 into the nucleus from the cytosol (Figure 7a). The ETME-mediated inhibition of LPS-induced NF-κB/p65 translocation into the nucleus was further detected by immunofluorescence imaging (Figure 7b). These results suggest that ETME exerts anti-inflammatory effects by inhibiting NF-κB signaling activation.

### 3.8. ETME Inhibited MAPK Phospohrylation

**Figure 8:**
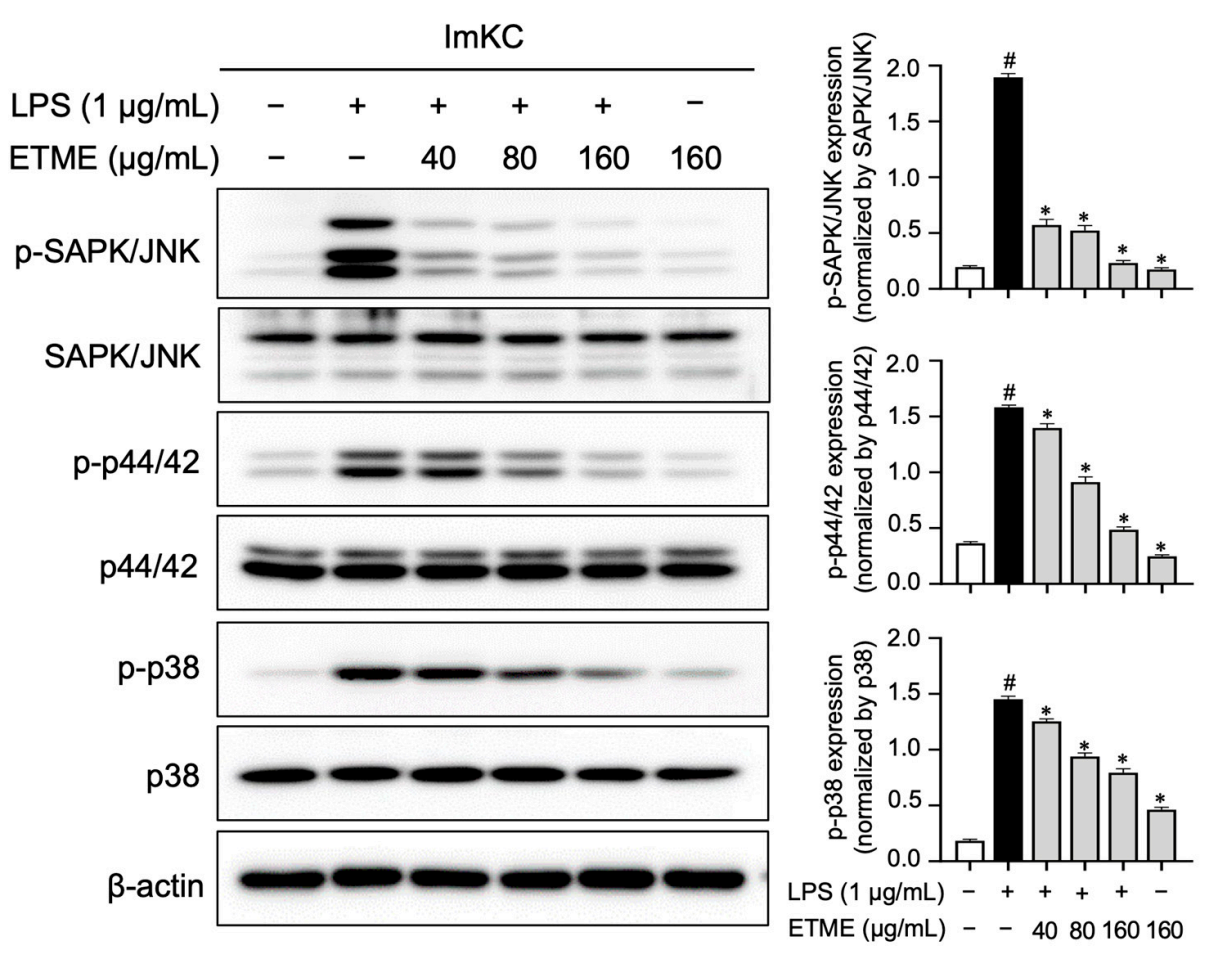
Inhibitory effects of ETME on mitogen-activated protein kinase (MAPK) signaling. The ImKCs were pretreated with ETME (40, 80, or 160 μg/mL) for 2 h, then stimulated with LPS (1 μg/mL) for 15 min. The expression of proteins associated with the MAPK signaling pathway (p38, p44/42, and SAPK/JNK) was detected by Western blotting, with β-actin as a loading control. The expression of phosphorylated (p-) protein (p-p38, p-p44/42, and p-SAPK/JNK) was normalized to that of the total-form protein (p38, p44/42, and SAPK/JNK). The data presented are the mean ± SEM of three independent experiments. Differences between groups were analyzed using the Mann–Whitney U test. # *p* < 0.05 vs. LPS-untreated group; * *p* < 0.05 vs. LPS-treated group.

MAPK pathways are also major inflammatory signaling pathways, which are activated and characterized by LPS-mediated phosphorylation [53,54]. The engagement of MAPK signaling pathways was investigated by measuring the ETME-mediated decrease in MAPK phosphorylation in LPS-treated ImKCs. The phosphorylation of MAPKs (p38, p44/42, and JNK) was markedly reduced by ETME treatment in a dose-dependent manner (Figure 8), suggesting that the anti-inflammatory effect is mediated by the suppression of MAPK activation in LPS-stimulated ImKCs.

## 4. Discussion

NO plays an important role in liver physiology and pathophysiology [55,56]. It is produced as a by-product when L-arginine is oxidized to citrulline by the action of three isomorphs: neuronal NOS (nNOS), iNOS, and endothelial NOS (eNOS). nNOS and eNOS are constitutively expressed. Although iNOS is not expressed under resting conditions, it is induced by immunological stimuli such as LPS [57]. Given the oxidative stress and pro-inflammatory nature of iNOS, and the beneficial role of iNOS inhibition in liver fibrosis, iNOS has been considered a potential therapeutic target for various diseases, including liver fibrosis [58,59], septic shock [60], and asthma [61]. Therefore, the inhibitory effect of ETME on NO production via the inhibition of LPS-induced iNOS expression in ImKCs suggests that ETME may be a therapeutic candidate for oxidative stress and inflammatory diseases in the liver, including liver fibrosis.

HO-1, an inducible enzyme, plays a cytoprotective role against oxidative stress by removing cytotoxic free heme and generating antioxidants [62,63]. Recent studies have revealed that it also exhibits anti-inflammatory activities against numerous inflammatory diseases. For example, rare HO-1 deficiencies in humans and animal models are characterized by high levels of chronic inflammation and increased sensitivity to oxidative stress [64,65,66,67]. Therefore, the immunomodulatory functions of HO-1 provide a promising therapeutic target for treating a broad range of oxidative and inflammatory diseases [62,63,68,69]. Here, ETME increased HO-1 expression in LPS-stimulated ImKCs (Figure 6a), and SnPP, an HO-1 inhibitor, significantly restored the ETME-induced reduction in NO and pro-inflammatory cytokines (Figure 5). These results suggest that ETME’s anti-inflammatory effects are closely related to its antioxidative properties, implying that ETME could be a valuable medication for treating various diseases via its cooperative regulatory effects on inflammation and oxidative stress.

The Keap1-Nrf2 pathway is a protective response to oxidative stress in macrophages [70]. Under homeostatic conditions, Keap1 tightly associates with Nrf2 and acts as a member of E3 ubiquitin ligase, which tightly regulates ubiquitination and proteasomal degradation of Nrf2 [71]. In response to oxidative stress, Nrf2 escapes ubiquitination by dissociating from Keap1, accumulating within the cell, translocating to the nucleus, and promoting its antioxidant transcription program [72]. Immunoblotting and immunofluorescence data revealed that Nrf2 accumulation in the nucleus was enhanced by the ETME treatment in the absence or presence of LPS (Figure 6). Cellular Nrf2 expression could be induced by enhancing its transcription and stabilizing it through the inhibition of its poly-ubiquitination. To clarify this, ML385 and brusatol, Nrf2 inhibitors that inhibit Nrf2 transcription [73] and stimulate its polyubiquitination [74], respectively, were treated and compared to the ETME-treated group. However, both ML385 and brusatol did not alleviate ETME-mediated NO inhibition in LPS-stimulated macrophages, indicating that the ETME-mediated accumulation of Nrf2 in the nucleus is not regulated by its transcription and polyubiquitination. Further study using other inhibitors, such as ascorbic acid, an electrophilic modifier of Keap1, or trigonelline, an inhibitor of nuclear translocation of Nrf2, could clarify the mechanism for the ETME-mediated nuclear increase in Nrf2 and subsequent antioxidant responses in macrophages.

The pharmacological activities of plant extracts are closely related to the pharmacological activities of various phytochemicals contained in the extracts. In this study, UPLC-Q-TOF-MS spectrometry analysis of ETME revealed that rosmarinic acid (component 9), uralenneoside (component 11), and acetyl-β-boswellic acid (component 20) were identified as the major components (Figure 1 and Table 1). Among these components, rosmarinic acid was found to exhibit both antioxidant [22] and anti-inflammatory effects [75]. In detail, rosmarinic acid elicits neuroprotection in ischemic stroke via Nrf2/HO-1 pathway [76] and ameliorates acute liver damage and fibrogenesis accompanied by enhanced Nrf2/HO-1 expression [77]. Rosmarinic acid is also known to inhibit LPS-induced NO production and iNOS in RAW264.7 macrophage via suppressing NF-κB signaling [78]. Also, rosmarinic acid attenuates the LPS-stimulated proinflammatory mediators such as TNF-α, IL-8, and iNOS through the inhibition of three MAPKs and NF-κB signaling in vascular smooth muscle cell [79]. Based on the high similarity between our results and previous reports on rosmarinic acid, it seems that the antioxidant and anti-inflammatory effects of ETME are mainly attributed to rosmarinic acid. Meanwhile, the known effects of these two components (uralenneoside and acetyl-β-boswellic acid) are insufficient to account for the antioxidant and anti-inflammatory pharmacological effects of ETME. Acetyl-β-boswellic acid was reported to have only limited anti-inflammatory property through the inhibition of 5-lipoxygenase and MAPKs [80,81], and no reports are available for the antioxidant and anti-inflammatory effects of uralenneoside. Since the effect of an extract is due to several constituents, additional studies to elucidate the antioxidant and anti-inflammatory properties of uralenneoside and acetyl-β-boswellic acid are required to clarify ETME-mediated effects.

## 5. Conclusions

This study elucidates the antioxidant and anti-inflammatory effects of a methanol extract of *E. tinifolia* and its underlying mechanisms of action. ETME significantly restored total GSH levels and suppressed NO and pro-inflammatory cytokine production. These inhibitions were mediated by the activation of the antioxidant Nrf2/HO-1 pathway and the inhibition of NF-κB translocation and MAPK phosphorylation. These results provide evidence supporting the traditional pharmacological efficacy reported for ETME, suggesting its potential application as a candidate capable of alleviating pathological conditions mediated by inflammation or oxidative stress.

## Figures and Tables

**Table 1:** Tentative identification of constituents in the ETME using UPLC-Q-TOF-MS spectrometry analysis. TML: traditional medicine library.

#	Observed RT (min)	Neutral Mass (Da)	Observed Neutral Mass (Da)	Observed (*m*/*z*)	Adducts	Component Name
1	0.54	504.16903	504.1691	503.1619	−H, +HCOO	Raffinose [28]
2	0.58	150.05282	150.0527	195.0509	+HCOO, −H	Pentose (TML)
3	4.43	242.09429	242.0938	241.0865	−H	Flavanthrinin [29]
4	4.48	536.20463	536.2039	581.2022	+HCOO	Schisantherin A [30]
5	4.60	610.15338	610.1547	609.1475	−H	Kaempferol-3,7-diglucoside [31]
6	4.86	594.15847	594.1591	593.1519	−H	Genistein-7,4′-di-O-β-D-glucoside [32]
7	5.03	286.08412	286.0831	285.0759	−H	Phyllodulcin [33]
8	5.50	314.07904	314.0787	313.0714	−H	Ermanin [34]
9	5.63	360.08452	360.0848	359.0775	−H	Rosmarinic acid [35]
10	5.75	312.06339	312.0631	357.0613	+HCOO, −H	2-Acetyl emodin (TML)
11	6.00	286.06887	286.0688	321.0399	+Cl	Uralenneoside (TML)
12	6.28	340.0583	340.0584	339.0511	−H	Versicolorin B [36]
13	6.47	374.10017	374.1001	373.0928	−H	Methyl rosmarinate [26]
14	6.60	354.07395	354.0741	353.0668	−H	5-Methoxysterigmatocystn [38]
15	6.77	336.027	336.0268	335.0195	−H	Rufescidride [39]
16	6.99	518.32435	518.3231	517.3159	−H	2-Hydroxyesculentic acid (TML)
17	8.07	576.12678	576.124	575.1167	−H	Procyanidin A2 [40]
18	13.71	256.24023	256.2403	255.233	−H	Methyl pentadecanote (TML)
19	15.67	510.17373	510.1744	555.1726	+HCOO	Globularinin (TML)
20	18.15	498.37091	498.3713	497.3641	−H, +HCOO	Acetyl-β-boswellic acid [41]
21	19.00	206.16707	206.1668	205.1595	−H	Longicamphenylone (TML)
22	19.57	416.36543	416.3664	461.3646	+HCOO	γ-Tocopherol [42]

## Data Availability

The data presented in this study are available in the article and Supplementary Material.

## Supplementary Materials

The supporting information can be downloaded at: https://www.mdpi.com/article/10.3390/antiox12101792/s1. Figure S1: HPLC chromatogram of Rosmarinic acid and ETME. Figure S2: Radical scavenging effect of rosmarinic acid.
