# Supplementary material for: Inhibitory Effects of Ehretia tinifolia Extract on the Excessive Oxidative and Inflammatory Responses in Lipopolysaccharide-Stimulated Mouse Kupffer Cells
Source: Antioxidants (Basel). 2023 Sep 22;12(10):1792. doi: 10.3390/antiox12101792 (PMC10604099; doi:10.3390/antiox12101792)
Supplement: Supplementary file 1 [file antioxidants-12-01792-s001.zip › antioxidants-2593782-supplementary.pdf]

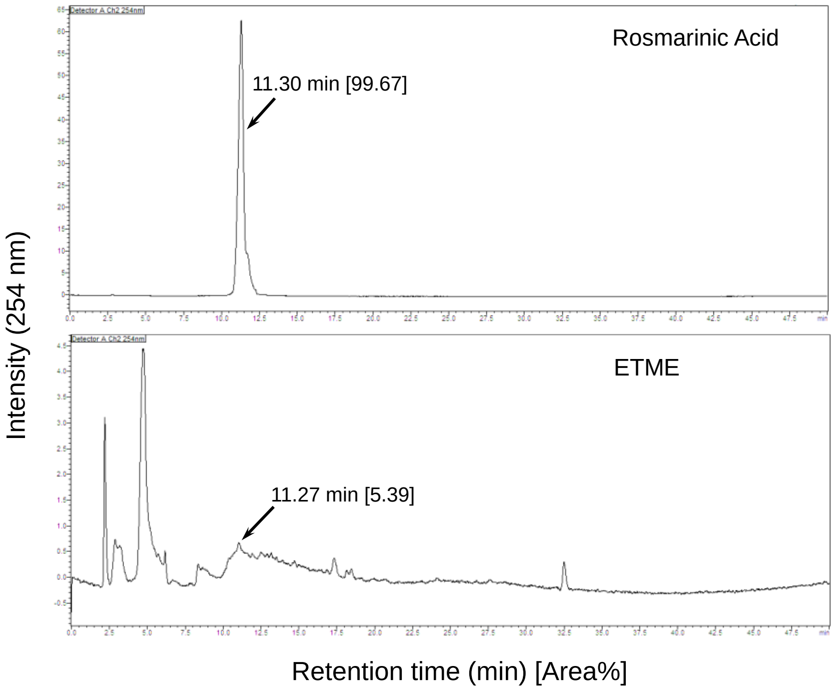

Supplementary figure S1. HPLC chromatogram of Rosmarinic acid and ETME. The results were analyzed using a HPLC system (Shimadzu Corp., Japan) with a SPD-20A UV/Vis detector, a LC-20AR solvent pump. Chromatography was achieved on an Atlantis T3 Column (4.6 mm x 250 mm, 5 μm) and monitored at 254 nm. Linear gradients at flow rate 1 ml/min with H_2O (A) and CH_3CN (B) were applied as follows: 0–40 min, 10 to 90% B, 40–50 min, 90% to 100% B.

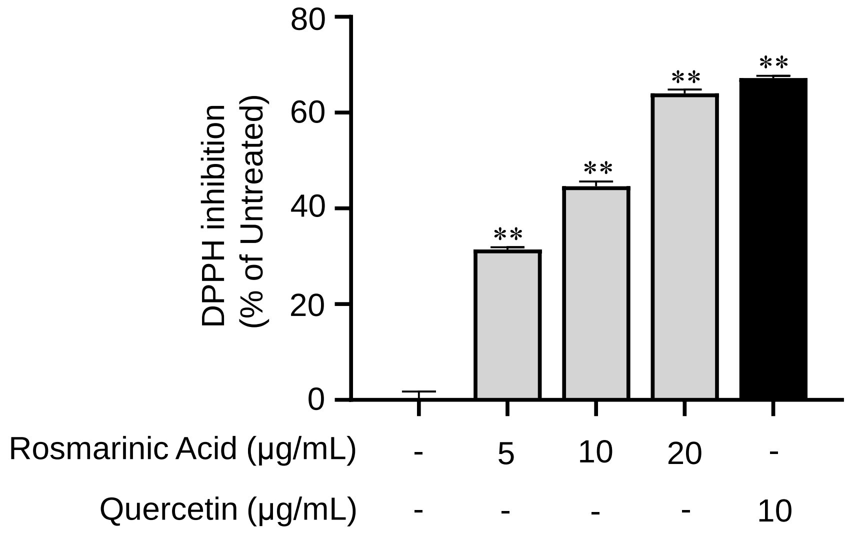

Supplementary figure S2. Radical scavenging effect of rosmarinic acid. The DPPH inhibition was expressed as % using the OD value of the experimental group and the value of 100% methanol solution (as a negative control). Quercetin was used as positive control.
